# Single- and dual-task gait performance and their diagnostic value in early-stage Parkinson's disease

**DOI:** 10.3389/fneur.2022.974985

**Published:** 2022-10-14

**Authors:** Xiaodan Zhang, Weinv Fan, Hu Yu, Li Li, Zhaoying Chen, Qiongfeng Guan

**Affiliations:** Department of Neurology, Hwa Mei Hospital, University of Chinese Academy of Sciences, Ningbo, China

**Keywords:** gait analysis, dual task, wearable sensors, Parkinson's disease, diagnosis

## Abstract

**Background:**

Gait parameters are considered potential diagnostic markers of Parkinson's disease (PD). We aimed to 1) assess the gait impairment in early-stage PD and its related factors in the single-task (ST) and dual-task (DT) walking tests and 2) evaluate and compare the diagnostic value of gait parameters for early-stage PD under ST and DT conditions.

**Methods:**

A total of 97 early-stage PD patients and 41 healthy controls (HC) were enrolled at Hwa Mei hospital. Gait parameters were gathered and compared between the two groups in the ST and DT walking test, controlling for covariates. Utilizing the receiver operating characteristic curve, diagnostic parameters were investigated.

**Results:**

In the ST walking test, significantly altered gait patterns could be observed in early-stage PD patients in all domains of gait, except for asymmetry (*P* < 0.05). Compared to the ST walking test, the early-stage PD group performed poorly in the DT walking test in the pace, rhythm, variability and postural control domain (*P* < 0.05). Older, heavier subjects, as well as those with lower height, lower level of education and lower gait velocity, were found to have a poorer gait performance (*P* < 0.05). Stride length (AUC = 0.823, sensitivity, 68.0%; specificity, 85.4%; *P* < 0.001) and heel strike angle (AUC = 0.796, sensitivity, 71.1%; specificity, 80.5%; *P* < 0.001) could distinguish early-stage PD patients from HCs with moderate accuracy, independent of covariates. The diagnostic accuracy of gait parameters under ST conditions were statistically noninferior to those under DT conditions(*P*>0.05). Combining all gait parameters with diagnostic values under ST and DT walking test, the predictive power significantly increased with an AUC of 0.924 (sensitivity, 85.4%; specificity, 92.7%; *P* < 0.001).

**Conclusion:**

Gait patterns altered in patients with early-stage PD but the gait symmetry remained preserved. Stride length and heel strike angle were the two most prominent gait parameters of altered gait in early-stage of PD that could serve as diagnostic markers of early-stage PD. Our findings are helpful to understand the gait pattern of early-stage PD and its related factors and can be conducive to the development of new diagnostic tools for early-stage PD.

## Introduction

Parkinson's disease (PD) is a common neurodegenerative disorder and represents a raising cause of disability worldwide and a growing burden on society (1). PD is characterized by bradykinesia, rigidity, and tremor; as the disease progresses, these symptoms worsen and result in severe disability. Therefore, it is crucial to diagnose PD as soon as possible in order to improve its clinical management and attempt to slow down its progression. Currently, the diagnosis of PD is primarily based on clinical evaluation. However, the limited accuracy and low repeatability of clinical evaluation make the early diagnosis of PD challenging, thereby increasing direct and indirect medical costs (2). New imaging examinations, such as positron emission tomography/computed tomography, can be used for the early diagnosis of PD but due to the radiation exposure risk, they are not generally applicable (3). In the past decade, numerous candidate biomarkers for the diagnosis of PD have been identified but most of them are limited in clinical practice due to their low accuracy (4). Consequently, it is crucial to develop safe, reliable, and effective clinical diagnostic markers to improve the clinical management of PD (5).

Gait impairment is one of the primary motor symptoms of PD and it will worsen as the disease progresses, even leading to falls and subsequent disability (6). In prodromal PD, studies have indicated that the nuclei and fibers involved in postural gait regulation could be impaired (7). Previous studies have suggested subtle changes in gait could be detected in prodromal PD, especially in rapid eye movement sleep behavior disorder (8–10). In addition, a previous study demonstrated that quantitative gait alterations could be observed ~4 years before PD diagnosis, indicating that certain gait parameters have the potential to serve as early diagnostic markers of PD (11). Mild gait disorders, such as reduced gait velocity, stride length, arm swing amplitude, greater interlimb asymmetry, and gait variability, may be one of the earliest indicators of PD (12–14). In early-stage PD, slower gait velocity and shorter stride length are indicative of bradykinesia, rigidity, and diminished motion range (13). Reduced heel height is associated with a dragging gait in PD patients (15). The greater variability and asymmetry of gait, reflect gait instability and the unilateral onset of PD (13, 16). In addition, an increasing number of studies have implemented the gait paradigm of PD patients under dual-task (DT), in which subjects were required to perform a cognitive task while walking (17–20). In DT walking test, when additional cognitive resources are mobilized for gait planning and management, PD patients can exhibit more severe gait impairment (17, 18, 21). These studies suggest that the DT gait test widens the gap between PD and healthy populations, indicating that it may be a viable method for investigating gait perturbations in PD and detecting early-stage PD.

Clinically, it can be difficult to observe these subtle gait changes with the naked eye, and traditional Gait Analysis requires large equipment that is not always available (13). With the development of technology, new gait analysis tools based on inertial sensors enable the quantitative detection of mild gait changes in PD patients and reduce evaluator discrepancies (22). The quantitative gait analysis can therefore be implemented in clinical practice and may contribute to the early diagnosis of PD (11). However, previous studies also have some limitations. First, though numerous spatiotemporal and dynamic characteristics have been studied in PD patients, the gait characteristics vary with no consistency across studies, the lack of control over covariates makes it difficult to compare gait parameters between studies, and it is still unclear which of the many gait parameters is best for the early diagnosis of PD (13, 23–26). To solve these problems, Lord et al. have proposed a structured approach to the measurement of gait in PD to standardize the study's quality, and the spatiotemporal gait characteristics of PD have been divided into five modal domains: pace, rhythm, variability, asymmetry, and postural control (23). Studies using structured gait measurement and controlling for covariates are needed to increase the comparability of different studies and explore diagnostic gait markers for early-stage PD. Second, most of the previous studies have focused on spatiotemporal gait parameters, while few studies have analyzed kinematic parameters in PD patients and the results are controversial in the limited studies (12, 27–29). Further studies validating changes in kinematic gait parameters in early-stage PD are needed. Third, it is unclear whether walking under DT conditions has a negative effect on kinematic parameters other than spatiotemporal gait parameters (30). Establishing the effect of DT on various gait parameters and their associated factors may aid in identifying risks associated with DT. Besides, though patients with early-stage PD present a more impaired gait pattern in the DT gait test, rare studies have investigated the advantage of using the DT walking test to diagnose early-stage PD (13).

Consequently, this study aims to: (1) evaluate the potential influences of confounders on standard measured gait parameters; (2) compare the gait performance between early-stage PD patients and healthy adults in a single task (ST) and DT walking test, controlling for covariates; and (3) evaluate and compare the diagnostic value of gait parameters for early-stage PD patients under ST and DT condition. Our findings may be helpful to understand the gait pattern of early-stage PD and its related factors and provide a low-cost, feasible, and effective method for early diagnosis of PD, accordingly allowing for early disease intervention.

## Materials and methods

### Participants

From September 2019 to December 2021, 97 patients with early-stage PD were enrolled at Hwa Mei hospital, University of Chinese Academy Of Sciences, with the following inclusion criteria: (1) diagnosis of PD according to Movement Disorder Society (MDS) criteria (31); (2) Hoehn and Yahr (H&Y) scale stages 1-2; (3) able to walk independently; (4) stable recent symptoms and medication. The following were the criteria for exclusion: (1) other diseases that may affect gait performance; (2) unable to comply with the doctor's instructions. A total of 41 healthy subjects from the community were enrolled in the healthy control (HC) group. The HC group matched the early-stage PD group in terms of age and gender, and the inclusion criteria were as follows: (1) no history of diseases that could impact gait performance, such as PD, cerebrovascular disease, depression, dementia, vestibular diseases, or orthopedic disease; and (2) able to comply with doctor's instructions.

The research was conducted in accordance with the Helsinki Declaration. All participants voluntarily participated and signed an informed consent form before the study. Hwa Mei hospital and the Chinese Academy of Sciences granted ethical approval for the research (approval number: PJ-NBEY-KY-2020-023-01).

### Clinical data collection

All participants' demographic characteristics were collected. The same specialist collected medical data and performed physical examinations on patients with early-stage PD. Part III of the Movement Disorder Society-Sponsored Revision of the Unified Parkinson's Disease Rating Scale (MDS-UPDRS III) was employed to assess the severity of motor symptoms. The Berg Balance Scale (BBS) and Mini-Balance Evaluation Systems Test (Mini-BEST) were used to assess balance function and fall risk. The H&Y scale was used to assess the severity of the disease, while the Activity of Daily Living Scale (ADL) was utilized to assess the quality of daily life. The Mini-Mental State Examination (MMSE) was used to assess cognitive function, whereas the Hamilton Depression Rating Scale-24 (HMAD) was used to assess depression. All PD patients were evaluated in the OFF state (the antiparkinsonian medication was stopped for 18 h).

### Gait evaluation

Using the JiBuEn^®^ gait analysis system, gait data was collected (32). This system consisted of shoes and modules with Micro-Electro-Mechanical System sensors on the waist, thigh, lower limb, and heel bottom of the shoe, and it transmitted motion data to a computer. The high-order low-pass filter and hexahedral calibration technique are employed in data preprocessing, which reduces high-frequency noise interference and installation errors produced by sensor devices. Moreover, the accumulative errors are also corrected based on the zero-correction algorithm. The final gait parameters are obtained by fusing acceleration data and posture, which is calculated using the quaternary complementary filtering technique. The validation of the JiBuEn^®^ system in measuring gait parameters has been evaluated (33).

All participants were required to complete two walking tests: (1) ST walking test: All participants walked in a straight line on a 10 m footpath at their preferred “natural” gait velocity, and gait parameters were collected during natural walking; (2) DT walking test: All participants walked in a straight line on the same 10 m footpath under DT. They were instructed to perform serial subtraction of 7 beginning with 100 while walking at their usual pace. During DT walking, they were instructed to focus on both tasks. Before the walking tests, all participants received one practice trial for walking under both ST and DT without data collection with the JiBuEn^®^.

Spatiotemporal gait parameters were determined as follows based on at least 40 steps: gait velocity (GV), stride length (SL), stride time, swing time, and stance time (23). The stance phase was calculated. Toe-off angle (TO) and heel strike angle (HS) were also obtained as kinematic parameters using this system. As reported, HS and TO are associated with postural instability in PD patients; consequently, we categorized them into the postural control domain (34). The variability of the left and right gait parameters was calculated separately and then combined to form the coefficient of variation (CV) (35). We calculated the variability of GV (CV-GV), SL (CV-SL), stride time (CV-stride time), swing time (CV-swing time), stance time (CV-stance time), TO (CV-TO), and HS (CV-HS). Using the asymmetry index (AI), the symmetry of SL (AI-SL), stride time (AI-stride time), swing time (AI-swing time), and stance time (AI-stance time) were evaluated (36).

### Statistical analysis

SPSS 26.0 software (IBM, Armonk, NY, USA) was used to analyse the data. The comparison of measured data between groups was evaluated by using the independent *t*-test for normally distributed data expressed as mean differences ± standard deviation (x ± s), and the Mann-Whitney *U* test for non-normally distributed data expressed as medians (interquartile ranges, IQRs). The χ^2^ test was used to evaluate the count data. The correction between GV and other gait parameters was analyzed using Spearman's correlation. The correlation values were considered very high (0.90–1.00), high (0.70–0.90), moderate (0.50–0.70), low (0.30–0.50), or negligible (0.00–0.30) (37). The level of significance was set to 0.05. In all analyses including gait parameters, the significance level was adjusted by Benjamini-Hochberg multiple testing correction with a prespecified false discovery rate of 0.05.

The Generalized Linear Mixed Model (GLMM) was used to analyse the data at two levels (level 1: task conditions, intra-individuals; Level 2: subjects, individuals) (38). To fit the model, task conditions (ST and DT) were assigned as repeated variables, and each gait parameter was used as a dependent variable. The initial model for gait parameters contained the following explanatory variables as fixed effects: grouping (e.g., early-stage PD group and HC group), task status (e.g., ST and DT), grouping ^*^ task status, and covariates (age, gender, education levels, height, weight, the score of MMSE and HAMD, with or without GV). Additionally, intercept and task status were regarded as random effects. GLMM was also used to control covariates in the comparison of groups. Using the regression coefficient test, the effects of grouping, task status, clinical parameters, and non-motor symptoms on gait parameters were analyzed.

The receiver operating characteristic (ROC) curve analysis was performed to evaluate the predictive performance of gait parameters using the pROC package of R language version 4.0.3 (39). The area under the curve (AUC) was calculated and compared using the bootstrap method with 2,000 iterations. Youden index was used to determine the optimal threshold for predicting early-stage PD. Figures were configured using Graph Pad Prism Software version 8.0.1.

## Results

### Demographic characteristics

This study included a total of 97 patients with early-stage PD. The mean disease duration of the early-stage PD group was 4.36 ± 4.50 years, the mean score of MDS-UPDRS III was 30.7 ± 14.41, and the median levodopa equivalent daily dose in the early-stage PD group was 309.38 (350) mg. In addition, 41 participants were assigned to the HC group. There were no statistically significant differences in age, gender, height, weight, or education level between the two groups (all *P*>0.05), but the score of MMSE score in the PD group was slightly lower than that of the HC group. In the early-stage PD group, the score of HAMD was higher than in the HC group (*P* < 0.05). Table 1 displays the clinical characteristics of all participants.

**Table 1:** Clinical characteristics of participants.

	**PD**	**HC**	***P***
N	97	41	
Age (years)	66.46 ± 9.20	62.49 ± 12.00	0.623
Male (%)	55 (56.70)	17 (41.50)	0.105
Height (cm)	163.00 (10.00)	165.00 (10.00)	0.362
Weight (kg)	62.00 (15.00)	65.00 (12.00)	0.180
Education (years)	6.00 (6.00)	9.00 (3.00)	0.278
MMSE	27.00 (5.00)	28.00 (3.00)	**0.012**
HADM	6.00 (8.00)	3.00 (7.00)	**0.005**
Duration of PD (years)	4.36 ± 4.50		
LEDD (mg)	309.38 (350.00)		
H-Y stage 1 (%)	22 (22.68)		
MDS-UPDRS III	30.70 ± 14.41		
BBS	52.74 ± 5.68		
Mini-BEST	24.57 ± 3.38		
ADL	96.74 ± 8.97		

Values are expressed as n (%), mean ± standard deviation or median (interquartile range). Independent t test, the χ^2^ test, or the Mann–Whitney U-test were performed for comparisons.

P < 0.05 were considered statistically significant.

Bold values highlight the significant difference.

PD, Parkinson's Disease; HC, healthy control; MMSE, Mini-Mental State Examination; HAMD, Hamilton Depression Rating Scale; LEDD:Levodopa Equivalent Daily Dose; H-Y stage, Hoehn and Yahr stage; MDS-UPDRS III, Movement Disorder Society-Sponsored Revision of the Unified Parkinson's Disease Rating Scale part III; BBS, the Berg Balance Scale; Mini-BEST, Mini-Balance Evaluation Systems Test; ADL, Activity of Daily Living Scale.

### Influences of confounders on gait performance

In all subjects, SL, stance time, stance phase, TO and HS were strongly correlated with GV in both walking tests (*P* < s0.001). Stride time, CV-SL, AI-SL, and CV-TO showed a moderate correlation with GV in all walking tests, although only a low or negligible correlation with GV could be observed in other parameters (Supplementary Table 1). After controlling for covariates, GV was significantly correlated with all gait parameters except for AI-GV and AI-stance time (*P* < 0.05, Table 2; Supplementary Table 2).

**Table 2:** Results of the generalized linear mixed models for each gait parameter controlling for gait velocity.

	**Task (ref ST)**	**Group (ref controls)**	Group*Task	**GV**
	β **(95%CI)**	β **(95%CI)**	β **(95%CI)**	β **(95%CI)**
SL (m)	0.028 (0.007,0.05)	**−0.087 (−0.12,−0.055)**	−0.006 (−0.031,0.019)	**0.634 (0.573,0.696)**
CV-Swing time (%)	−0.071 (−2.444,2.302)	−2.581 (−5.306,0.143)	0.973 (−1.844,3.790)	**−12.748 (−18.210,−7.287)**
Stride time (s)	0.019 (−0.023,0.062)	**−0.148 (−0.199,−0.097)**	0.017 (−0.034,0.067)	**−0.697 (−0.804,−0.590)**
Stance time (s)	0.012 (−0.019,0.043)	**−0.113 (−0.151,−0.075)**	0.012 (−0.024,0.048)	**−0.642 (−0.718,−0.566)**
Swing time (s)	0.007 (−0.004,0.018)	**−0.032 (−0.044,−0.02)**	0.004 (−0.009,0.017)	**−0.065 (−0.091,−0.039)**
Stance phase (%)	−0.066 (−0.221,0.352)	**−0.937 (−1.450,−0.424)**	0.023 (−0.314,0.359)	**−13.645 (−14.571,−12.719)**
CV-GV (%)	−2.48 (−5.45,0.49)	−1.857 (−6.363,2.649)	−0.818 (−4.354,2.717)	**−10.303 (−16.198,−4.408)**
CV-SL (%)	−0.011 (−2.212,2.191)	−0.043 (−1.853,1.767)	−0.442 (−3.070,2.186)	**−11.439 (−14.967,−7.91)**
CV-Stride time (%)	−0.72 (−4.184,2.745)	−4.569 (−8.687,−0.451)	2.267 (−1.850,6.383)	**−12.897 (−20.532,−5.261)**
CV-Stance time (%)	−0.178 (−1.734,1.377)	−1.362 (−3.212,0.489)	0.774 (−1.072,2.621)	**−3.947 (−7.541,−0.352)**
AI-GV (%)	**−5.538 (−8.367,−2.709)**	2.446 (−4.482,9.375)	0.154 (−3.141,3.449)	−4.553 (−15.180,6.075)
AI-Swing time (%)	−2.182 (−7.729,3.365)	−3.828 (−10.705,3.048)	2.895 (−3.683,9.474)	**−22.859 (−36.160,−9.558)**
AI-Stride time (%)	−2.723 (−8.622,3.175)	−8.746 (−15.444,−2.047)	4.271 (−2.741,11.282)	**−13.485 (−26.216,−0.754)**
AI-Stance time (%)	−0.535 (−2.966,1.896)	−1.323 (−4.324,1.677)	0.899 (−1.984,3.782)	−5.762 (−11.604,0.080)
AI-SL (%)	−0.234 (−5.035,4.568)	0.618 (−3.432,4.668)	−1.431 (−7.164,4.302)	**−19.583 (−27.274,−11.892)**
TO (°)	0.64 (−77.569,78.85)	0.023 (−93.867,93.914)	−0.320 (−94.199,93.559)	**22.451 (18.169,26.733)**
HS (°)	0.489 (−0.194,1.173)	**−3.471 (−5.025,−1.917)**	−0.288 (−1.086,0.509)	**20.257 (17.762,22.751)**
CV-TO (%)	−0.193 (−2.791,2.405)	−2.917 (−5.599,−0.236)	0.955 (−2.142,4.053)	**−19.419 (−24.459,−14.378)**
CV-HS (%)	0.399 (−2.499,3.298)	−1.350 (−4.062,1.362)	0.413 (−3.045,3.871)	**−17.390 (−22.639,−12.142)**

The Generalized Linear Mixed Model was used to analyze the effects of group, task, group^*^ task.

The models were controlled by gender, age, height, weight, education level, score of Mini-Mental State Examination and Hamilton Depression Rating Scale, gait velocity.

All of the P-values were corrected using Benjamini–Hochberg multiple testing correction.

P < 0.05 were considered statistically significant and highlighted in bold.

CI, confidence intervals; β, beta; ref, reference; Group^*^Task: The interaction between group and task; ST, single task; GV, gait velocity; SL, stride length; TO, toe-off angle; HS, heel strike angle; CV, coefficient of variation; AI, asymmetry index.

In patients with early-stage PD, male subjects had a longer SL, a greater CV-GV, CV-ST, CV-stance time, and AI-ST compared to female subjects (*P* < 0.05). In addition, as the education year lengthens, a longer SL, faster GV, bigger TO and HS, shorter ST, smaller AI-GV and AI-SL could be observed (*P* < 0.05). However, the score of MMSE and HAMD only had a weak effect on gait parameters in patients with early-stage PD (*P* < 0.05), as shown in Table 3; Supplementary Table 3.

**Table 3:** Influences of clinical features on the gait parameters in early-stage PD group.

	**Intercept**	**Task**	**Age**	**Gender**	**Height**	**Weight**	**Education**	**HADM**	**MMSE**	**MDS–UPDRS III**
		**(ref ST)**	**(y)**	**(ref female)**	**(cm)**	**(kg)**	**(y)**	
GV (m/s), β	**1.364**	**−0.112**	**−0.004**	0.030	0.002	−0.003	**0.011**	−0.004	−0.007	**−0.006**
SL (m), β	**1.640**	**−0.048**	**−0.006**	**0.083**	0.002	**−0.003**	**0.009**	−0.002	**−0.010**	**−0.007**
CV-Swing time (%), β	**69.565**	**2.431**	0.076	3.274	**−0.306**	−0.001	−0.127	0.050	−0.062	−0.053
Stride time (s), β	**1.215**	**0.114**	0.001	0.050	−0.001	0.001	−0.006	0.003	−0.001	0.001
Stance time (s), β	0.689	**0.096**	0.001	0.026	−0.001	0.002	**−0.006**	**0.003**	0.001	0.001
Swing time (s), β	**0.466**	**0.018**	−0.001	0.018	0.001	−0.001	−0.001	0.001	−0.001	−0.001
Stance phase (%), β	**55.474**	**1.607**	0.044	−0.878	−0.013	**0.076**	**−0.128**	**0.074**	0.089	**0.058**
CV-GV (%), β	**73.646**	**−2.112**	0.097	**4.187**	−0.242	0.019	−0.319	−0.016	−0.260	0.102
CV-SL (%), β	14.318	0.949	**0.136**	0.989	−0.027	−0.030	−0.178	0.090	0.087	**0.109**
CV-Stride time (%), β	**84.951**	**3.189**	0.143	**6.772**	**−0.405**	−0.034	−0.260	0.044	−0.043	−0.123
CV-Stance time (%), β	**52.311**	**1.104**	0.043	**2.732**	**−0.216**	−0.023	−0.051	0.022	−0.057	**−0.060**
AI-GV (%), β	**177.328**	**−4.540**	0.169	5.986	**−0.833**	0.260	**−1.174**	0.130	−0.304	0.107
AI-Swing time (%), β	**122.038**	3.731	0.256	7.416	**−0.680**	−0.056	−0.525	0.202	0.036	−0.102
AI-Stride time (%), β	**115.241**	3.500	0.318	**11.098**	**−0.690**	−0.052	−0.506	0.123	0.132	−0.152
AI-Stance time (%), β	**55.145**	1.167	0.118	3.418	**−0.291**	−0.056	−0.204	0.080	0.045	−0.051
AI-SL (%), β	33.040	0.985	**0.265**	4.131	−0.239	−0.016	**−0.650**	0.113	0.331	**0.199**
TO (°), β	**99.358**	**−2.214**	**−0.345**	0.719	−0.103	**−0.129**	**0.495**	−0.129	−0.258	**−0.206**
HS (°), β	**87.382**	**−2.087**	**−0.253**	2.848	−0.184	−0.070	**0.275**	−0.107	−0.171	**−0.243**
CV-TO (%), β	11.641	**2.929**	0.116	1.698	−0.014	0.005	−0.193	0.083	−0.022	**0.107**
CV-HS (%), β	32.701	**2.746**	0.132	3.006	−0.109	−0.004	−0.168	0.046	−0.026	**0.094**

The Generalized Linear Mixed Model was used to analyze the influence of clinical features on the gait parameters in early-stage PD group.

P < 0.05 were considered statistically significant and highlighted in bold.

β, beta; ref, reference; ST, single task; GV, gait velocity; SL, stride length; TO, toe-off angle; HS, heel strike angle; CV, coefficient of variation; AI, asymmetry index; MMSE, Mini-Mental State Examination; HAMD, Hamilton Depression Rating Scale; MDS-UPDRS III, Movement Disorder Society-Sponsored Revision of the Unified Parkinson's Disease Rating Scale part III.

### Gait parameters in DT walking test compared with ST walking test

**Figure 1:**
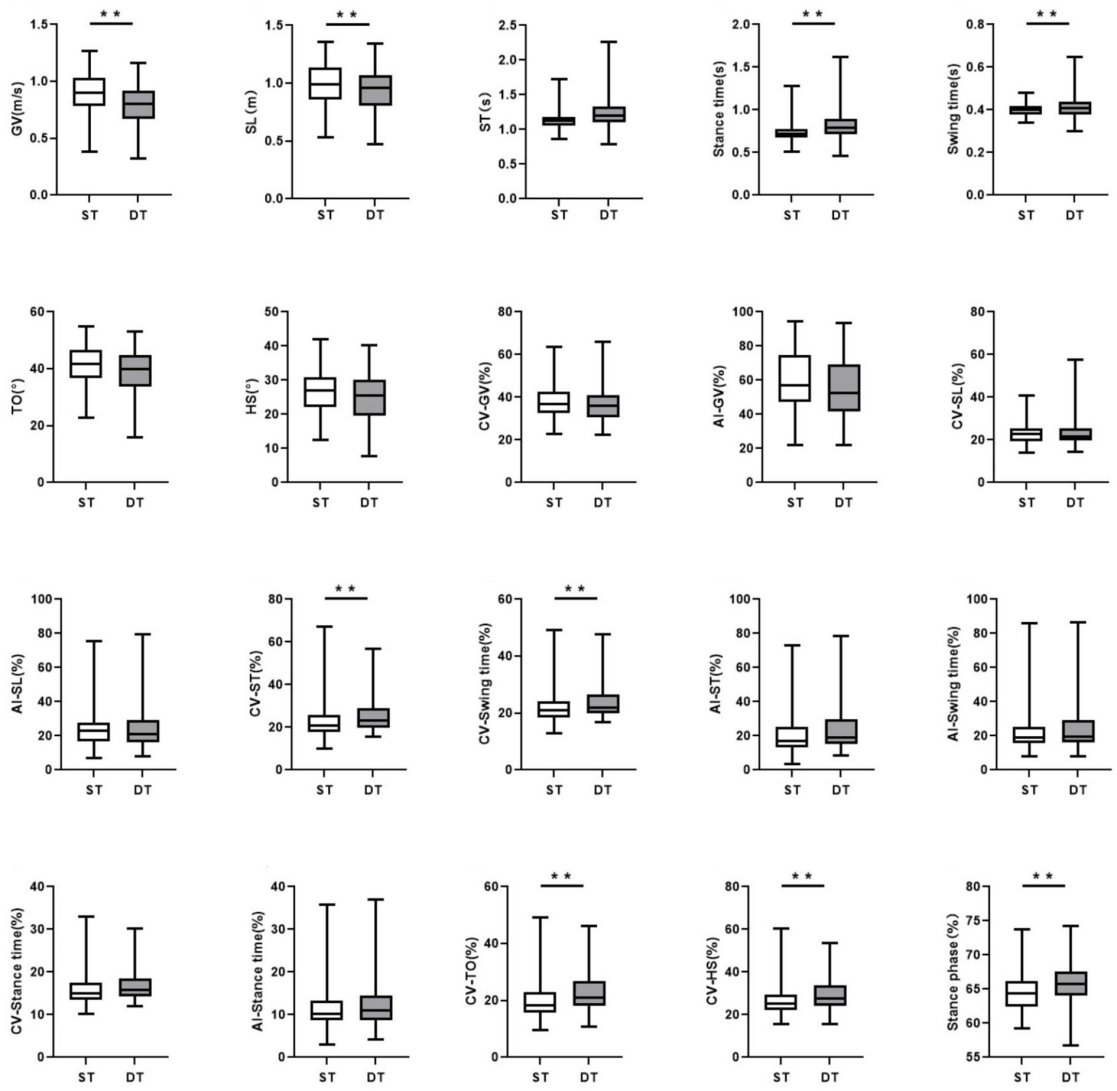
The comparisons of gait characteristics of Parkinson's Disease in single task walking and dual task walking. ST, single task; DT, dual task; PD, Parkinson's Disease; HC, healthy control; GV, gait velocity; SL, stride length; ST, stride time; TO, toe-off angle; HS, heel strike angle; CV, coefficient of variation; AI, asymmetry index. All of the P-values were corrected using Benjamini–Hochberg multiple testing correction. ^**^p ≤ 0.05.

**Figure 2:**
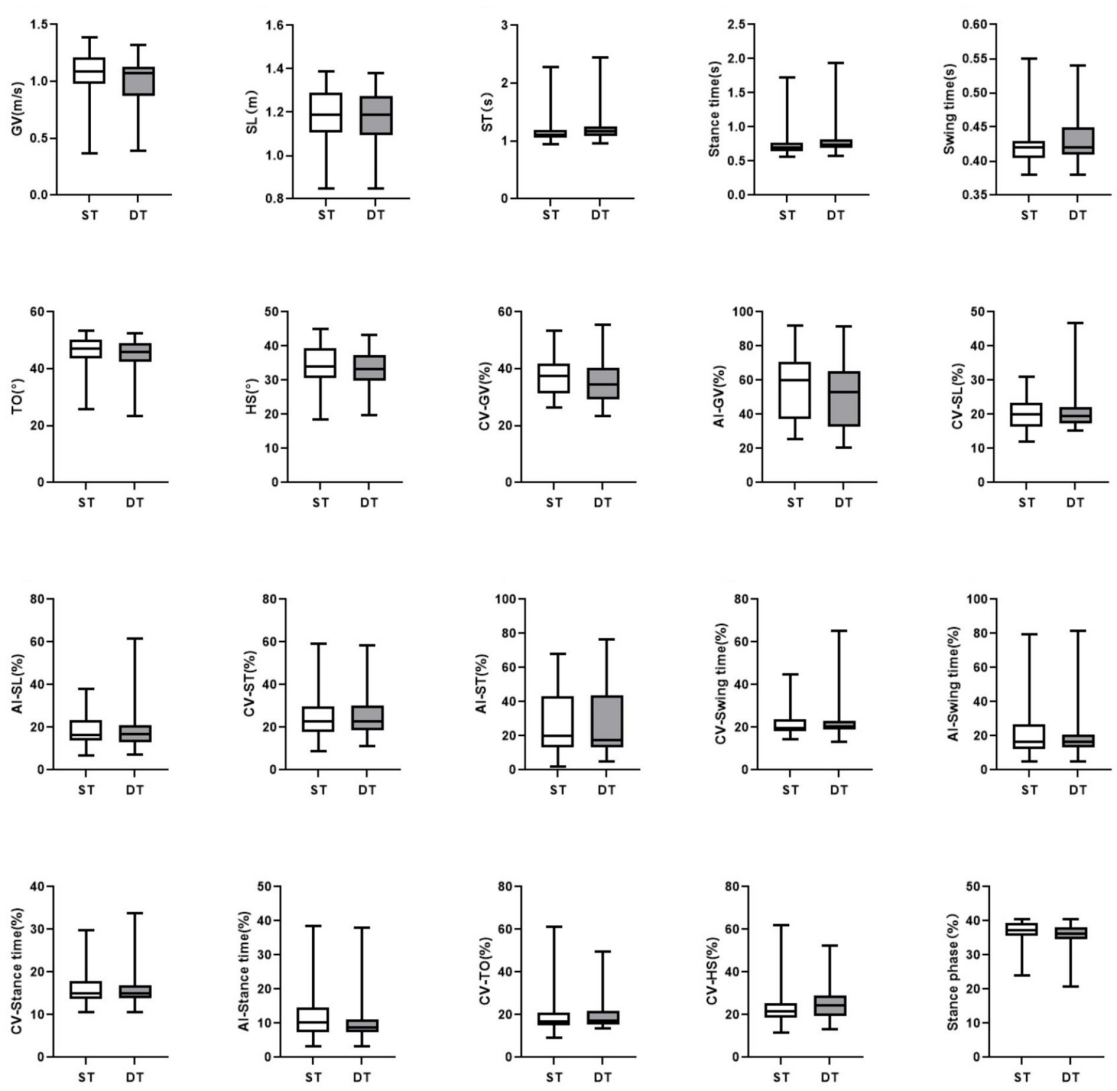
The comparisons of gait characteristics of healthy adults in single task walking and dual task walking. ST, single task; DT, dual task; PD, Parkinson's Disease; HC, healthy control; GV, gait velocity; SL, stride length; ST, stride time; TO, toe-off angle; HS, heel strike angle; CV, coefficient of variation; AI, asymmetry index. All of the P-values were corrected using Benjamini–Hochberg multiple testing correction. **p ≤ 0.05.

In the DT walking test, patients with early-stage PD demonstrated significantly impaired pace, rhythm, variability, and postural control domain than in the ST walking test (*P* < 0.05, Figure 1). After controlling for covariates (age, gender, height, weight, levels of education, MMSE scores, HAMD scores, and UPDRS-III scores), all the differences remained significant (*P* < 0.05, Table 3; Supplementary Table 3). However, compared to ST, no significant changes in gait parameters were observed in the HC group under DT conditions (*P* > 0.05, Figure 2).

### Gait parameters in the early-stage PD patients compared with healthy controls

In both ST and DT walking tests, patients with early-stage PD exhibited a slower GV, a shorter SL, a bigger stance phase, a greater AI-SL and CV-SL, a smaller TO and HS, and a greater CV-HS than HCs (*P* < 0.05). After controlling for GV, compared with HC group, a shorter SL, longer stance phase, and smaller HS could be observed in the early-stage PD group in both walking tests, while a smaller swing time only in the ST walking test (*P* < 0.05), as shown in Table 4.

**Table 4:** Comparison of gait characteristics of patients with early-stage PD and healthy controls.

	**ST**					**DT**				
	**HC**	**PD**	***P***	**Adj*.P***	**Adj*.P'***	**HC**	**PD**	***P***	**Adj*.P***	**Adj*.P'***
**Pace**	
GV (m/s)	1.06 ± 0.20	0.89 ± 0.19	**<0.001**	**0.005**	**0.005**	0.99 ± 0.19	0.78 ± 0.19	**<0.001**	**<0.001**	**<0.001**
SL (m)	1.19 ± 0.14	0.99 ± 0.18	<0.001*	**<0.001**	**<0.001**	1.17 ± 0.12	0.93 ± 0.20	<0.001*	**<0.001**	**<0.001**
CV-Swing time (%)	19.49 (5.36)	20.99 (5.16)	0.558	0.903	**0.155**	20.23 (4.09)	21.98 (6.63)	**0.044**	0.749	0.567
**Rhythm**
Stride time (s)	1.11 (0.12)	1.12 (0.13)	0.872	0.114	**<0.001**	1.17 (0.15)	1.20 (0.23)	0.366	0.610	**<0.001**
Stance time (s)	0.70 (0.13)	0.72 (0.11)	0.290	0.255	**<0.001**	0.74 (0.12)	0.79 (0.18)	0.087	0.910	**<0.001**
Swing time (s)	0.42 (0.03)	0.40 (0.04)	0.004*	**<0.001**	**0.004**	0.42 (0.04)	0.41 (0.06)	0.054	0.740	**<0.001**
Stance phase (%)	62.67 (3.72)	64.32 (3.84)	0.006*	0.280	**0.006**	63.67 (3.55)	65.71 (3.50)	<0.001*	0.084	**0.001**
**Variability**
CV-GV (%)	37.64 (10.50)	36.63 (9.99)	0.631	0.913	**0.026**	35.38 ± 7.84	36.18 ± 7.31	0.676	0.343	0.381
CV-SL (%)	20.08 ± 4.21	22.96 ± 5.28	**0.005**	0.122	0.496	19.38 (4.82)	21.79 (5.73)	**0.004**	0.640	0.733
CV-stride time (%)	22.95 (11.71)	20.77 (7.95)	0.208	0.508	0.241	22.73 (11.55)	23.37 (9.07)	0.692	0.641	0.347
CV-Stance time (%)	15.01 (3.96)	15.09 (3.87)	0.783	0.877	0.381	15.00 (3.00)	15.89 (4.18)	0.299	0.613	0.733
**Asymmetry**
AI-GV (%)	59.80 (33.48)	58.00 (28.20)	0.256	0.662	0.894	53.00 (32.54)	52.54 (27.54)	0.189	0.486	0.804
AI-Swing time (%)	16.67 (13.14)	18.75 (8.54)	0.195	0.951	0.640	16.67 (7.00)	19.51 (13.00)	**0.010**	0.747	0.733
AI-Stride time (%)	20.0 (29.16)	17.19 (12.14)	0.262	0.281	0.175	17.74 (30.07)	19.14 (14.39)	0.530	0.249	0.226
AI-Stance time (%)	10.15 (7.03)	10.29 (4.35)	0.430	0.877	0.567	8.70 (3.58)	10.96 (5.72)	0.053	0.853	0.958
**Postural control**
AI-SL (%)	16.28 (9.37)	22.92 (10.98)	**0.004**	0.131	0.638	16.67 (8.02)	21.05 (13.01)	**0.004**	0.613	0.638
TO (°)	45.97 ± 5.76	40.97 ± 7.02	**<0.001**	**0.027**	0.825	44.99 ± 5.57	38.83 ± 7.58	**<0.001**	**0.020**	0.880
HS (°)	34.07 ± 6.05	26.67 ± 6.68	<0.001*	**<0.001**	**<0.001**	33.09 ± 5.51	24.66 ± 6.73	<0.001*	**<0.001**	**<0.001**
CV-TO (%)	16.81 (6.01)	18.26 (7.25)	0.085	0.692	**0.383**	17.34 (6.28)	21.02 (8.61)	**0.006**	0.303	0.057
CV-HS (%)	21.63 (7.13)	25.15 (7.51)	**0.007**	0.850	0.733	24.59 (9.59)	27.44 (9.66)	0.010	0.174	0.401

Variables are expressed as mean ± standard deviation or median (interquartile range). Independent t test or the Mann–Whitney U-test were performed for comparisons.

Adj.P, P-value were controlled for gender, age, height, weight, education level, score of Mini-Mental State Examination and Hamilton Depression Rating Scale.

Adj.P', P-value were controlled for gender, age, height, weight, education level, score of Mini-Mental State Examination and Hamilton Depression Rating Scale, GV.

All of the P-values were corrected using Benjamini–Hochberg multiple testing correction. Bold values highlight the significant difference.

* The significant difference after adjusting for GV.

ST, single task; DT, dual task; PD, Parkinson's Disease; HC, healthy control; GV, gait velocity; SL, stride length; TO, toe-off angle; HS, heel strike angle; CV, coefficient of variation; AI, asymmetry index.

After controlling for covariates (age, gender, height, weight, levels of education, the scores of MMSE and HAMD), some of the differences were no longer statistically significant, including stance phase, CV-SL, AI-SL, and CV-HS in the ST walking test (*P*>0.05). During the DT walking test, only the differences of GV, SL, TO, and HS remained significant after controlling for covariates (*P* < 0.05), as shown in Table 4. The interaction between task status and grouping was statistically significant in HS and TO (*P* < 0.05, Table 5; Supplementary Table 4).

**Table 5:** Results from the generalized linear mixed models for each gait parameter not controlling for gait velocity.

	**Intercept**	**Task (ref ST)**	**Group (ref controls)**	Group*Task
	β **(95%CI)**	β **(95%CI)**	β **(95%CI)**	β **(95%CI)**
GV (m/s)	**1.678 (0.686, 2.671)**	**−0.072 (−0.107**, **−0.037)**	**−0.130 (−0.200**, **−0.060)**	−0.039 (−0.081, 0.003)
SL (m)	**1.540 (0.665, 2.414)**	−0.017 (−0.044, 0.009)	**−0.171 (−0.231**, **−0.111)**	−0.031 (−0.062, 0.001)
CV-Swing time (%)	**58.934 (22.532, 95.335)**	0.849 (−1.581, 3.279)	−0.971 (−3.676,1.734)	1.472 (−1.445, 4.389)
Stride time (s)	0.826 (−0.206, 1.857)	**0.070 (0.002, 0.137)**	−0.061 (−0.140,0.017)	0.044 (−0.037,0.125)
Stance time (s)	0.254 (−0.605, 1.112)	**0.058 (0.001, 0.115)**	−0.034 (−0.095, 0.027)	0.038 (−0.031, 0.106)
Swing time (s)	**0.403 (0.223, 0.583)**	0.011 (−0.002,0.025)	**−0.022 (−0.035**, **−0.010)**	0.006 (−0.010, 0.022)
Stance phase (%)	**48.617 (33.635, 63.600)**	**1.051 (0.476, 1.626)**	0.852 (−0.182,1.885)	0.558 (−0.132, 1.247)
CV-GV (%)	**68.461 (32.271, 104.650)**	**−1.736 (−2.836**, **−0.636)**	−0.846 (−3.424, 1.733)	−0.414 (−1.735, 0.906)
CV-SL (%)	15.994 (−8.085, 40.074)	0.815 (−1.393, 3.024)	1.380 (−0.569, 3.328)	0.007 (−2.645, 2.658)
CV-Stride time (%)	82.906 (33.096, 132.715)	0.211 (−3.269, 3.692)	−3.073 (−7.132, 0.986)	2.772 (−1.405, 6.950)
CV-Stance time (%)	47.913 (24.733, 71.092)	0.107 (−1.447, 1.661)	−0.930 (−2.730, 0.871)	0.929 (−0.936, 2.794)
AI-GV (%)	**146.536 (48.215, 244.856)**	**−5.209 (−7.920**, **−2.498)**	2.151 (−4.662, 8.963)	0.332 (−2.922, 3.587)
AI-Swing time (%)	101.891 (13.291, 190.491)	−0.531 (−6.106, 5.043)	−1.058 (−7.878, 5.763)	3.791 (−2.900, 10.483)
AI-Stride time (%)	132.064 (50.680, 213.449)	−1.750 (−7.610, 4.111)	−7.143 (−13.708, −0.577)	4.799 (−2.235, 11.834)
AI-Stance time (%)	51.493 (13.196, 89.789)	−0.119 (−2.532, 2.293)	−0.621 (−3.550, 2.308)	1.125 (−1.771, 4.021)
AI-SL (%)	25.984 (−24.341, 76.310)	1.180 (−3.597, 5.957)	3.176 (−1.024, 7.376)	−0.663 (−6.397, 5.071)
TO (°)	**91.091 (57.836, 124.346)**	−0.980 (−1.974, 0.013)	**−3.144 (−5.464**, **−0.825)**	**−1.200 (−2.392**, **−0.008)**
HS (°)	**79.249 (47.393 to 111.105)**	**−0.973 (−1.838 to** **−0.109)**	**−6.981 (−9.266 to** **−4.695)**	**−1.035 (−2.066 to** **−0.003)**
CV-TO (%)	6.194 (−29.564, 41.952)	1.209 (−1.503, 3.922)	−0.730 (−3.596, 2.137)	1.696 (−1.563, 4.956)
CV-HS (%)	24.711 (−11.438, 60.860)	1.655 (−1.283, 4.593)	0.643 (−2.219, 3.506)	1.071 (−2.459, 4.601)

The Generalized Linear Mixed Model was used to analyze the effects of group, task, group^*^ task.

The models were controlled by gender, age, height, weight, education level, score of Mini-Mental State Examination and Hamilton Depression Rating Scale.

All of the P-values were corrected using Benjamini–Hochberg multiple testing correction.

P < 0.05 were considered statistically significant and highlighted in bold.

CI, confidence intervals; β, beta; ref, reference; Group^*^Task: the interaction between group and task; ST, single task; GV, gait velocity; SL, stride length; TO, toe-off angle; HS, heel strike angle; CV, coefficient of variation; AI, asymmetry index.

After additional controlling for both covariates and GV, significant differences in SL, stride time, swing time, stance time, and TO could be observed between the early-stage PD group and HC group in all walking tests, while a difference of CV-GV could only be observed between groups in the DT walking test (*P* < 0.05), as shown in Table 4. No interaction between task status and grouping could be observed after further controlling for GV (*P* > 0.05, Table 2; Supplementary Table 2).

### Diagnostic value of gait parameters for early-stage PD patients under ST and DT

**Figure 3:**
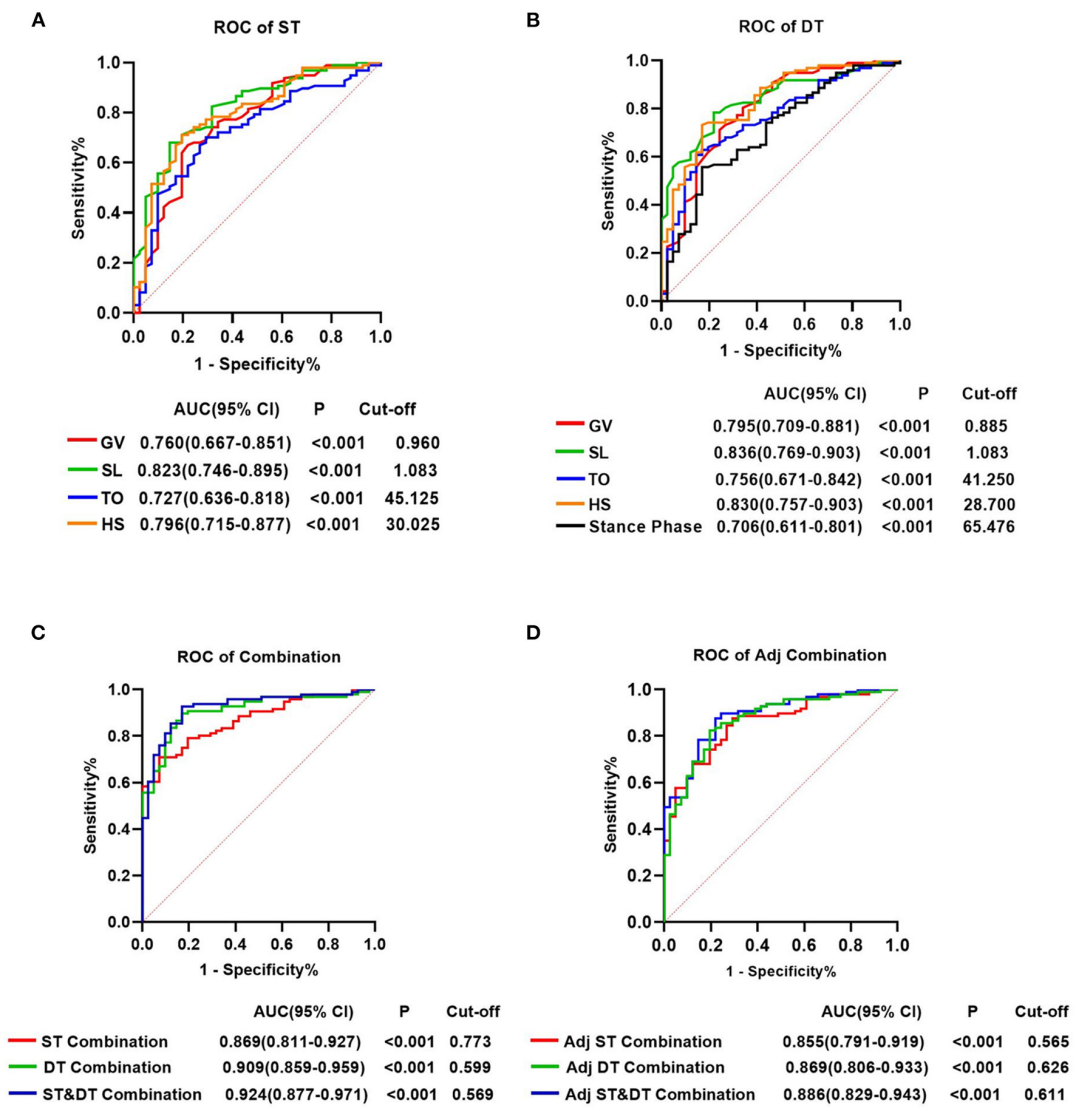
Receiver operating characteristics analysis plots for gait parameters distinguishing the individuals with early-stage Parkinson's Disease and healthy controls. **(A)** GV, SL, TO, and HS for identifying patients with PD in the single task walking test. **(B)** GV, SL, TO, HS and stance phase for identifying patients with PD in the single task walking test. **(C)** ST-Combination, receiver operating characteristics (ROC) analysis for the combination of GV, SL, TO, HS, swing time, stance phase, CV-HS, CV-SL, and AI-SL under ST; DT-Combination, DT-Combination, ROC analysis for the combination of GV, SL, TO, HS, stance phase, CV-SL, AI-SL, CV-TO, CV-HS, CV-Swing time and AI-Swing time under DT; ST&DT-Combination, ROC analysis for the combination of all gait parameters with diagnostic values under ST and DT. **(D)** Adj ST-Combination, ROC analysis for the combination of GV, SL, swing time, stance phase, and HS under ST. Adj DT-Combination, ROC analysis for the combination of GV, SL, stance phase, and HS under DT. Adj ST& DT-Combination, ROC analysis for the combination of ST-GV, ST-SL, ST-swing time, ST-stance phase, ST-HS, DT-GV, DT-SL, DT-stance phase, and DT-HS. AUC, area under the curve; CI, confifidence interval; cut-off, cut-off point; ST, single task; DT, dual task; GV, gait velocity; SL, stride length; TO, toe-off angle; HS, heel strike angle; CV, coefficient of variation; AI, asymmetry index.

For all gait parameters with significant differences between the early-stage PD and HC groups, ROC curve analysis was performed to determine their diagnostic utility. Nine gait parameters from the ST walking test and eleven gait parameters from the DT walking test had significant predictive values for early-stage PD (*P* < 0.05, Supplementary Table 5). The AUC value indicated that GV, SL, TO, and HS in both walking tests, and the Stance phase in the DT walking test had a moderate ability to distinguish early-stage PD from HC (AUCs > 0.700, *P* < 0.001) (Supplementary Table 5; Figure 3). At a cut-off of 1.083, the AUC value, sensitivity and specificity of SL were 0.823, 68.0% and 85.4%, respectively, in the ST walking test (*P* < 0.001, Supplementary Table 5; Figure 3). Following HS with an AUC of 0.796 in the ST walking test, at a threshold of 30.025, the sensitivity of HS was 71.1%, whereas the specificity was 80.5% (*P* < 0.001, Supplementary Table 5; Figure 3).

In DT walking test, the AUC value of all gait parameters increased, but not significantly (*P* > 0.05, Figure 3, Supplementary Tables 5, 6). SL demonstrated the most accurate predictive performance in the DT walking test, with an AUC value increased to 0.836, a sensitivity of 78.4%, and a specificity of 78.0% at the cut-off of 1.083, followed by HS with an increased AUC value of 0.830, a sensitivity of 73.2%, and a specificity of 82.9% at the cut-off of 28.700 (*P* < 0.001, Supplementary Table 5; Figure 3).

In addition, we attempted to combine the 9 diagnostic gait parameters in the ST walking test and discovered that the accuracy of prediction increased significantly, with an AUC of 0.869, a sensitivity of 70.8%, and a specificity of 92.7%. Combining the 11 diagnostic gait parameters under DT increased the predicted AUC to 0.909, with a sensitivity of 89.7% and a specificity of 82.9%. After combining all gait parameters with diagnostic values under ST and DT, the predictive power significantly increased compared with the combination of diagnostic parameters under ST, with an AUC increased to 0.924 (*P* < 0.001, Supplementary Tables 5, 6; Figure 3).

After adjusting for GV, 5 gait parameters from the ST walking test and 4 gait parameters from the DT walking test had significant predictive values for early-stage PD (*P* < 0.05, Supplementary Table 5). Interestingly, in both walking tests, after controlling for GV, the predictive power of the combined diagnostic parameters was statistically noninferior to that of the parameters without adjusting for GV (*P* > 0.05, Supplementary Tables 5, 6; Figure 3).

## Discussion

This cross-sectional, single-center, observational study aimed to identify gait parameters with a high degree of accuracy for early diagnosis of PD and to comprehend the gait pattern of early-stage PD patients in the ST and DT walking tests. Our results showed that: (1) demographic covariates and the score of HAMD, and GV could impact various gait parameters of PD; (2) In the DT walking test, the early-stage PD group demonstrated impaired pace, rhythm, variability, and postural control domain compared to the ST walking test. (3) SL and HS could distinguish early-stage PD and HC, independent of differences in GV; (4) The diagnostic accuracy of gait parameters increased, but not significantly, under DT condition as compared with those under ST. The diagnostic accuracy of the gait parameters significantly increased when ST and DT walking tests were combined.

### Influences of GV and confounders on gait performance

A recent systematic review has suggested that the spatiotemporal gait parameters and joint kinematics decreased at slower speeds (24). Particularly, for the older individuals, when they walked slower, the cadence and step length decreased (24). Consequently, the lower spontaneous walking speed of patients with early-stage PD may impact gait parameters, leading to an overestimation of pathological gait impairment (40). In this study, lower GV was highly associated with shorter SL, longer stance time, stride time, stance phase, smaller TO, and HS in all walking tests, in line with previous studies (40–42). A previous study has suggested that in patients with PD, the variability of stride time and swing time were independent of gait speed (43). In line with the previous study, the correlation between GV and CV-GV, CV-stride time, and CV-stance time, was low to negligible in this study, indicating the increased gait variability in PD was disease-related, and not simply a consequence of bradykinesia (43). Future research is needed to investigate the interaction of the central structure or function with the variability of gait in early-stage PD.

Non-motor symptoms of PD are reported to be associated with gait disturbances in PD (25, 26). Interestingly, this study suggested that cognitive function didn't have a significant influence on the gait performance of patients with early-stage PD, except for a minor impact on SL, inconsistent with previous studies (25, 44). We analyzed that the early-stage PD patients enrolled in this study were not suffered with severe cognitive impairment, and MMSE might not be sensitive enough to assess the mild cognitive impairment. To be considered, however, there are limitations of the MMSE in detecting attention and executive function responsible for gait performance in PD (45, 46). Future research using more precise assessments, such as extensive neuropsychological tests, is needed. As reported, depression was associated with gait disturbances in PD, including a lower GV and greater variability of stride time (26, 47). However, in this study, after controlling for the score of MDS-UPDRS III, only a mild influence of the HAMD score on stance time and stance phase could be observed in patients with early-stage PD. We attribute it to the fact that the higher score of HAMD was associated with poorer motor symptoms in PD patients (48). In addition, we only enrolled early-stage PD patients, while the majority of these patients did not have depression (31). Future studies should investigate the relationship between depression, motor symptoms and gait performance.

Consistent with the previous studies, we found demographic factors could impact the gait performance in subjects with early-stage PD, necessitating adjustment for these variables in order to standardize the study's quality and investigate the robust diagnostic markers of PD (49, 50). Among these, weight and education level are controllable variables. A previous study of healthy adults has shown that being overweight had a negative effect on gait performance, as evidenced by a shorter SL, a longer stance time, and a reduction in postural stability (51). Our study also showed that weight had a slight effect on SL and TO. Particularly, this study revealed that the year of education could improve gait performance in the pace, rhythm, asymmetry, and postural control domains, with the GV increasing by 0.011 m/s for each additional year of education. It can be explained by the contribution of education to increased cognitive reserve, which is linked to both milder motor deficits and cognitive impairment (52, 53). Controllable variables, such as weight and education, should be investigated in greater depth in the future and can be used to design future effective treatments to improve the gait pattern of people with PD (54).

### Influences of DT on gait performance of early-stage PD

Under DT, the walking task and the cognitive task compete for the limited information processing resources, resulting in a decline in task performance (55). Consistently with previous studies, patients with early-stage PD in this study demonstrated worse gait performance in the DT walking test in four domains, including pace, rhythm, variability, and postural control, when compared to the ST walking test (17–20). Overall, the influence of DT on the variability domains was significant across the biggest number of variables and the influence was highest compared to other domains in our study, in line with previous research (18). Reduced movement automaticity and increased conscious control can explain the phenomenon (55, 56). In addition, this study added some new findings: in the DT walking test, the variability of TO and HS were greater in the early-stage PD compared with ST, and a smaller TO and HS could be observed after further controlling for covariates. TO and HS are measured at the beginning or end point of the swing phase and can reflect the foot clearance and dragging gait in PD patients (57). A recent work, using a word-color interference test as the cognitive task, also suggested significant reductions in lower limb kinematics during toe-off and heel-strike could be observed in PD patients in DT walking when compared to ST walking (58). However, a previous study using forward digit span as the cognitive task suggested that TO was adversely affected by DT in PD patients, while HS was not (27). We attribute the difference in the results to the different complexity of the cognitive task and the different walking speed in the two studies, which will impact the performance of DT walking (21, 41). In future studies, the effects of DT on PD gait should be investigated in greater depth, which will facilitate the development of DT training to improve DT gait performance in patients with PD (59).

### Gait parameters in the early-stage PD patients compared with healthy controls

In line with previous studies, this study revealed that patients with early-stage PD presented impaired pace, rhythm, variability, and postural control domains of gait in the ST walking test, and an impaired pace, variability, asymmetry, and postural control domain in the DT walking test (12, 14, 19). Among these, gait variability was disease-related, and not significantly associated with GV according to previous research (43). Greater gait variability suggested increased conscious control, decreased automaticity, increased gait instability, and the beginning of impaired gait control (18). Due to the significant influence of DT on the variability of gait, the risk of falls in PD patients under DT should be considered and avoided (60). The regulation of steps is impaired in PD patients, and the asymmetry of gait can be a sensitive measure of gait instability (61). However, this study revealed that the gait symmetry remained preserved in early-stage PD, which is not consistent with previous studies (62, 63). We attribute the disparity to more advanced patients with PD were enrolled in previous studies. Consistent with our hypothesis, previous studies involving patients within H&Y stages 1-2 showed that the gait symmetry was not significantly altered in early-stage PD (12, 64). Future research is needed to examine how asymmetrical gait pattern varies with disease progression, and investigate the relationship between gait symmetry and symmetrical function of the motor cortex, the supplementary motor cortex and dopaminergic circuit in patients with early-stage PD to verify our hypothesis.

After controlling for demographic covariates and scores of MMSE and HAMD, differences in the TO, HS, SL and GV between the early-stage PD and HC groups remained statistically significant in both walking tests. The acquisition of the pace domain of gait is simple, so the GV and SL have been routinely measured in prior research (13). GV and SL reflect the bradykinesia and amplitude control of PD, both of them are dopa-responsiveness and change with disease progression (13). In line with previous studies, we discovered that SL was the most prominent parameter of altered gait in patients with early-stage PD under both ST and DT conditions (12, 13, 64). While lower GV is not unique to patients with PD, many other diseases including Alzheimer's disease can reduce GV, and GV may also be affected by age (13).

In line with previous studies, the HS was smaller in the early-stage PD group than in the HC group during both the ST and DT walking tests in this study (15, 27–29). A previous study suggested in patients with PD, the DT condition increased the attention required for joint flexion, extension, and muscle strength of the ankle (27). This study extended previous findings in showing that the DT gait test widened the gap of HS between the early-stage PD population and the HC, suggesting a more dragging gait when walking under DT conditions. Therefore, even early-stage PD patients should avoid performing complex cognitive tasks while walking on uneven terrain. Particularly, after adjusting for GV, the difference in HS and SL between groups was still significant, indicating these two parameters were disease-related. While in previous studies, the changes in TO in patients with early-stage PD remain controversial (12, 28, 29, 61). We attribute it to the inclusion of PD patients with different spontaneous GV and various stages in these studies (12, 28, 29, 61). In this study, TO was smaller in the early-stage PD group, but after adjusting for GV, the difference was not significant, consistent with previous research enrolling early-stage PD patients (15). This result indicated that altered TO in patients with early-stage PD was due to the slower spontaneous GV. Further research on TO and HS in early-stage PD under different speeds is required to explain the disparity in results.

### Diagnostic gait markers of early-stage PD

Recently, a growing number of studies aimed to distinguish PD patients from healthy individuals using gait features (65, 66). However, the classification accuracy for older adults and early-stage PD can be much more difficult than for advanced patients, as the gait impairment in PD patients worsens with progression (6). Based on the ROC curve analysis, 9 parameters in the ST waking test and 11 parameters in the DT walking test had predictive values for early-stage PD, especially SL, GV, TO, and HS had a moderate predictive value (AUC > 0.700). When the predictive parameters under both ST and DT conditions were combined, the AUC for early-stage PD prediction increased to 0.924, suggesting a combination of DT and gait analysis by wearable sensors could conduce to the early diagnosis of PD. While after adjusting for GV, HS, SL, swing time and stance phase had predictive values for early-stage PD. Interestingly, after combining these parameters, the diagnostic value of the combined markers was non-inferior to that of combined gait parameters not adjusting for GV. This finding is important because these disease-related markers controlled the influence of GV, making it easy to compare between studies, thus these gait parameters can be candidate gait markers for the early diagnosis of PD (24).

### Strengths and limitations of this study

The strengths of this study can be summarized as follows: (1) Using wearable sensors and controlling for covariates, we performed a comprehensive analysis of the gait impairment in early-stage PD patients compared with HC in ST and DT walking tests. (2) We extended previous studies by investigating the changes in kinematic gait parameters in early-stage PD under ST and DT conditions. (3) We compared the diagnostic value of gait parameters to distinguish early-stage PD from HC under ST and DT conditions.

As reported, dopaminergic treatment improves certain aspects of gait, including GV, SL, and foot dynamics (67–69). In addition, improved DT walking can be observed in patients in the ON state compared to those in the OFF state (68). Levodopa can also improve depression in a proportion of patients with PD (70). This study aimed to understand gait pattern of early-stage PD and its related factors, so we assessed PD patients in the OFF condition to exclude the influence of levodopa on gait performance, DT and other potential confounders. However, the limitation should be considered in that the different gait parameters' responsiveness to levodopa could not be evaluated to investigate their diagnostic values for PD. Consequently, the results of the study may not transfer to the ON stage of medication administration. In particular, patients with early-stage PD are mostly in the ON state, as they usually have a good response to dopaminergic medications. It is inconvenient to stop the antiparkinsonian medication to reveal OFF state before performing gait analysis in clinical practice, so the applicability of this potential paradigm to support the diagnosis of patients with early-stage PD is limited. In the future, the gait parameters of early-stage PD patients in both ON and OFF states should be investigated.

This study also has some other limitations. First, the participants were recruited from a single center, leading to potential selection biases. However, the consecutive recruitment and the large sample size of this study decreased the biases. Second, the additional information of the AUC is limited without the training set and testing set. So the results of our study could not be used for the diagnosis of PD in clinical practice yet. Future multi-center studies recruiting a larger sample of subjects should be conducted to collect more gait data for validation and tests. Third, the study was a cross-sectional study, while longitudinal data were unavailable, limiting the study of pathological gait signatures of PD. Fourth, the reliability of the ST and DT gait measures of PD patients could not be provided in this study, due to both walking tests were only performed once.

## Conclusion

In conclusion, the gait pattern altered in patients with early-stage PD, but the gait symmetry remained preserved. PD gait impairments may be exacerbated by modifiable factors such as DT, weight gain, and low education level. Gait parameters could distinguish early-stage PD patients from healthy controls. Among these, SL, and HS were the two most prominent gait parameters and had moderate predictive values for early-stage PD. Combining gait parameters under ST and DT can improve the accuracy of early-stage PD diagnosis and facilitate early intervention. Our findings contribute to understanding the gait pattern in patients with early-stage PD gait, are helpful in the future designs of effective treatments of gait impairment in PD and can be conducive to the development of new diagnostic tools for early-stage PD. Further multi-center, longitudinal studies are needed to evaluate the evolution of PD gait patterns and determine the diagnostic value of gait parameters for early-stage PD.

## Data availability statement

The raw data supporting the conclusions of this article will be made available by the authors, without undue reservation.

## Ethics statement

The studies involving human participants were reviewed and approved by the Ethics Committee of Hwa Mei Hospital, University of Chinese Academy of Science. The patients/participants provided their written informed consent to participate in this study.

## Author contributions

XZ contributed to the collecting clinical data, statistical analysis, and drafting the manuscript. WF, HY, and LL contributed to the recruitment of subjects and recording clinical data. QG and ZC revised the manuscript and conceived and supervised the work. All authors contributed to the article and approved the submitted version.

## Funding

This study was supported by Zhejiang Provincial Public Service and Application Research Foundation (Grant Nos. LGF20H090007 and LGF21H090009) and Ningbo Medical Key Discipline (Grant No. 2022-B12).

## Conflict of interest

The authors declare that the research was conducted in the absence of any commercial or financial relationships that could be construed as a potential conflict of interest.

## Publisher's note

All claims expressed in this article are solely those of the authors and do not necessarily represent those of their affiliated organizations, or those of the publisher, the editors and the reviewers. Any product that may be evaluated in this article, or claim that may be made by its manufacturer, is not guaranteed or endorsed by the publisher.
