# Corrigendum: Single- and dual-task gait performance and their diagnostic value in early-stage Parkinson's disease

**DOI:** 10.3389/fneur.2023.1334223

**Published:** 2023-11-17

**Authors:** Xiaodan Zhang, Weinv Fan, Hu Yu, Li Li, Zhaoying Chen, Qiongfeng Guan

**Affiliations:** Department of Neurology, Hwa Mei Hospital, University of Chinese Academy of Sciences, Ningbo, China

**Keywords:** gait analysis, dual task, wearable sensors, Parkinson's disease, diagnosis

In the published article, there was an error in the Funding statement. The Zhejiang Provincial Public Service and Application Research Foundation had incorrect grant numbers. The correct Funding statement appears below.

This study was supported by Zhejiang Provincial Public Service and Application Research Foundation (Grant Nos. LGF20H090007 and LGF21H090009) and Ningbo Medical Key Discipline (Grant No. 2022-B12).

The authors apologize for this error and state that this does not change the scientific conclusions of the article in any way. The original article has been updated.

