# Supplementary material for: Single- and dual-task gait performance and their diagnostic value in early-stage Parkinson's disease
Source: Front Neurol. 2022 Oct 14;13:974985. doi: 10.3389/fneur.2022.974985 (PMC9615249; doi:10.3389/fneur.2022.974985)
Supplement: Supplementary file 1 [file Data_Sheet_1.docx]

Supplemental Table 1. Correlations between gait velocity and gait parameters

|  | ST | | DT | |
| --- | --- | --- | --- | --- |
|  | Correlation Coefficient | *P* | Correlation Coefficient | *P* |
| SL(m) | **0.889** | <0.001 | **0.801** | <0.001 |
| CV-Swing time(%) | -0.232 | 0.006 | -0.395 | <0.001 |
| Stride time(s) | -0.571 | <0.001 | -0.583 | <0.001 |
| Stance time(s) | **-0.728** | <0.001 | **-0.700** | <0.001 |
| Swing time(s) | 0.080 | 0.352 | -0.028 | 0.746 |
| Stance phase(%) | **-0.890** | <0.001 | **-0.893** | <0.001 |
| CV-GV(%) | -0.267 | 0.002 | -0.364 | <0.001 |
| CV-SL(%) | -0.537 | <0.001 | -0.516 | <0.001 |
| CV-Stride time(%) | -0.048 | 0.576 | -0.271 | 0.001 |
| CV-Stance time(%) | 0.014 | 0.868 | -0.140 | 0.103 |
| AI-GV(%) | -0.214 | 0.012 | -0.280 | 0.001 |
| AI-Swing time(%) | -0.308 | <0.001 | -0.345 | <0.001 |
| AI-Stride time(%) | -0.034 | 0.696 | -0.210 | 0.013 |
| AI-Stance time(%) | -0.103 | 0.232 | -0.154 | 0.071 |
| AI-SL(%) | -0.527 | <0.001 | -0.516 | <0.001 |
| TO(°) | **0.822** | <0.001 | **0.772** | <0.001 |
| HS(°) | **0.880** | <0.001 | **0.806** | <0.001 |
| CV-TO(%) | -0.509 | <0.001 | -0.547 | <0.001 |
| CV-HS(%) | -0.504 | <0.001 | -0.471 | <0.001 |

The Spearman’s correction was used to analyze the correction between gait velocity and other gait parameters. Correlation Coefficient value>0.700 were considered high and highlighted in bold. ST, single task; DT, dual task; SL, stride length; TO, toe-off angle; HS, heel strike angle; CV, coefficient of variation; AI, asymmetry index.

Supplemental Table 2. Results of the generalized linear mixed models for each gait parameter controlling for gait velocity

|  | Model | Intercept | Task(ref ST) | Group(ref controls) | Group*Task | GV | Df |
| --- | --- | --- | --- | --- | --- | --- | --- |
|  | F | β(95%CI) | F | F | F | F |  |
| SL(m) | 82.787 | 0.311(-0.145, 0.768) | **13.380** | **25.444** | 0.220 | **417.415** | 266 |
| CV-Swing time(%) | **4.203** | **77.408(41.878, 112.938)** | 0.300 | 2.641 | 0.462 | **21.130** | 257 |
| Stride time(s) | **28.484** | **1.768(1.037, 2.499)** | **4.079** | **22.973** | 0.430 | **165.83** | 257 |
| Stance time(s) | **45.559** | **1.27(0.737, 1.802)** | 3.317 | **24.543** | 0.438 | **276.365** | 266 |
| Swing time(s) | **7.745** | **0.487(0.310, 0.664)** | **5.926** | **17.298** | 0.340 | **23.542** | 257 |
| Stance phase(%) | **130.535** | **72.217(64.807, 79.627)** | 0.656 | **11.369** | 0.018 | **842.805** | 256 |
| CV-GV(%) | **3.759** | **76.66(36.629, 116.69)** | **9.508** | 1.785 | 0.208 | **11.846** | 257 |
| CV-SL(%) | **9.219** | **33.919(12.420, 55.419)** | 0.114 | 0.111 | 0.110 | **40.746** | 257 |
| CV-Stride time(%) | **2.653** | **99.797(50.376, 149.218)** | 0.141 | 3.708 | 1.176 | **11.063** | 257 |
| CV-Stance time(%) | **1.789** | **52.398(28.948, 75.848)** | 0.177 | 1.327 | 0.682 | **4.676** | 257 |
| AI-GV(%) | **6.455** | **136.572(37.893, 235.252)** | **31.920** | 0.507 | 0.008 | 0.712 | 257 |
| AI-Swing time(%) | **2.573** | **134.692(46.921, 222.464)** | 0.171 | 0.566 | 0.751 | 11.454 | 257 |
| AI-Stride time(%) | **1.972** | **150.666(68.923, 232.409)** | 0.099 | 5.027 | 1.439 | 4.351 | 257 |
| AI-Stance time(%) | **1.469** | **59.442(20.887, 97.997)** | 0.012 | 0.395 | 0.377 | 3.772 | 257 |
| AI-SL(%) | **7.272** | **58.830(11.958, 105.702)** | 0.402 | 0.003 | 0.242 | **25.145** | 257 |
| TO(°) | 26.174 | 67.337(-15.273, 149.947) | 0.001 | 0.001 | 0.001 | **106.617** | 257 |
| HS(°) | **56.493** | **49.735(27.669, 71.800)** | 2.211 | **20.781** | 0.506 | **255.744** | 257 |
| CV-TO(%) | **10.602** | **31.991(0.258,63.723)** | 0.121 | 4.569 | 0.369 | **57.546** | 256 |
| CV-HS(%) | **9.750** | **48.031(15.419,80.644)** | 0.444 | 0.940 | 0.055 | **42.570** | 256 |

The Generalized Linear Mixed Model was used to analyze the effects of group, task, group* task.The models were controlled by gender, age, height, weight, education level, score of Mini-Mental State Examination and Hamilton Depression Rating Scale, gait velocity. *P* < 0.05 were considered statistically significant and highlighted in bold. CI, confidence intervals; β, beta; ref, reference; Group*Task: the interaction between group and task; ST, single task; GV, gait velocity; SL, stride length; TO, toe-off angle; HS, heel strike angle; CV, coefficient of variation; AI, asymmetry index; df, degree of freedom.

Supplemental Table 3. Influences of clinical features on the gait parameters in PD group

|  | Model | Task(ref ST) | Age(y) | Male(%) | Height(cm) | Weight(kg) | Education(y) | HADM | MMSE | UPDRS III | Df |
| --- | --- | --- | --- | --- | --- | --- | --- | --- | --- | --- | --- |
|  | **F** | **F** | **F** | **F** | **F** | **F** | **F** | **F** | **F** | **F** |  |
| GV(m/s) | **13.896** | **77.238** | **4.292** | 0.424 | 0.282 | 3.441 | **8.163** | 2.643 | 1.918 | **22.501** | 174 |
| SL(m) | **11.210** | **25.121** | **10.644** | **4.386** | 0.372 | **3.991** | **8.040** | 1.765 | **6.253** | **38.223** | 174 |
| CV-Swing time(%) | **2.446** | **8.558** | 1.049 | 3.680 | **6.022** | 0.001 | 0.791 | 0.378 | 0.125 | 1.351 | 174 |
| Stride time(s) | **2.958** | **14.887** | 0.001 | 1.890 | 0.172 | 0.810 | 3.697 | 3.419 | 0.006 | 0.270 | 174 |
| Stance time(s) | **3.625** | **15.860** | 0.167 | 0.717 | 0.167 | 1.850 | **4.805** | **4.422** | 0.050 | 1.847 | 174 |
| Swing time(s) | **3.030** | **16.981** | 1.516 | 3.645 | 0.001 | 0.423 | 0.353 | 0.316 | 0.388 | 3.843 | 174 |
| Stance phase(%) | **9.501** | **54.354** | 1.673 | 1.349 | 0.034 | **6.868** | **4.329** | 1.090 | **4.089** | **8.979** | 174 |
| CV-GV(%) | **6.097** | **32.194** | 1.221 | **4.283** | 2.674 | 0.062 | 3.553 | 0.028 | 1.549 | 3.503 | 174 |
| CV-SL(%) | **3.898** | 1.534 | **6.729** | 0.668 | 0.090 | 0.425 | 3.091 | 2.469 | 0.487 | **11.228** | 174 |
| CV-Stride time(%) | **2.754** | **6.717** | 1.924 | **8.102** | **5.413** | 0.139 | 1.697 | 0.156 | 0.031 | 3.701 | 174 |
| CV-Stance time(%) | **2.606** | **4.056** | 0.833 | **6.492** | **7.580** | 0.321 | 0.317 | 0.192 | 0.267 | **4.291** | 174 |
| AI-GV(%) | **6.146** | **32.211** | 0.626 | 1.483 | **5.368** | 1.937 | **8.139** | 0.312 | 0.358 | 0.652 | 174 |
| AI-Swing time(%) | **2.071** | 3.435 | 1.924 | 3.043 | **4.790** | 0.121 | 2.173 | 1.006 | 0.007 | 0.792 | 174 |
| AI-Stride time(%) | **2.663** | 3.051 | 3.879 | **8.914** | **6.434** | 0.133 | 2.640 | 0.486 | 0.118 | 2.321 | 174 |
| AI-Stance time(%) | **2.179** | 1.895 | 2.310 | 3.677 | **4.997** | 0.692 | 1.859 | 0.905 | 0.060 | 1.116 | 174 |
| AI-SL(%) | **3.557** | 0.341 | **5.548** | 2.537 | 1.590 | 0.027 | **8.967** | 0.847 | 1.528 | **8.137** | 174 |
| TO(°) | **12.509** | **34.252** | **25.710** | 0.211 | 0.813 | **4.684** | **14.307** | 3.030 | 2.554 | **24.023** | 174 |
| HS(°) | **13.359** | **41.845** | **15.690** | 3.766 | 2.937 | 1.557 | **5.010** | 2.361 | 1.267 | **37.790** | 174 |
| CV-TO(%) | **2.929** | **8.655** | 2.610 | 1.054 | 0.014 | 0.006 | 1.945 | 1.094 | 0.017 | **5.780** | 174 |
| CV-HS(%) | **2.673** | **6.532** | 3.203 | 3.140 | 0.764 | 0.003 | 1.401 | 0.325 | 0.022 | **4.261** | 174 |

The Generalized Linear Mixed Model was used to analyze the influence of clinical features on the gait parameters in early-stage PD group. *P* < 0.05 were considered statistically significant and highlighted in bold. ref, reference; ST, single task; GV, gait velocity; SL, stride length; TO, toe-off angle; HS, heel strike angle; CV, coefficient of variation; AI, asymmetry index; MMSE, Mini-Mental State Examination; HAMD, Hamilton Depression Rating Scale; UPDRS III, Movement Disorder Society-Sponsored Revision of the Unified Parkinson's Disease Rating Scale part III; Df, degree of freedom.

Supplemental Table 4. Results from the generalized linear mixed models for each gait parameter not controlling for gait velocity

|  | Model | Task(ref ST) | Group(ref controls) | Group*Task | Df |
| --- | --- | --- | --- | --- | --- |
|  | F | F | F | F |  |
| GV | **17.728** | **74.63** | **18.5** | 3.403 | 257 |
| SL | **17.728** | **74.630** | **18.500** | 3.403 | 257 |
| CV-Swing time | **11.973** | **16.638** | **35.696** | 3.623 | 257 |
| ST | **1.936** | **4.580** | 0.033 | 0.988 | 257 |
| Stance time | **4.867** | **19.905** | 1.167 | 1.155 | 257 |
| Swing time | **5.499** | **19.772** | 0.236 | 1.184 | 257 |
| Stance phase | **13.831** | **57.630** | **4.307** | 2.533 | 256 |
| CV-GV | **5.242** | **7.467** | **13.271** | 0.596 | 267 |
| CV-SL | **7.311** | **33.600** | 0.692 | 0.382 | 257 |
| CV-ST | **4.018** | 1.478 | 2.613 | 0.001 | 257 |
| CV-Stance time | 1.545 | 2.269 | 0.938 | 1.708 | 257 |
| AI-GV | 1.368 | 1.456 | 0.328 | 0.962 | 257 |
| AI-Swing time | **6.787** | **37.241** | 0.446 | 0.04 | 257 |
| AI-ST | 1.380 | 0.645 | 0.073 | 1.245 | 257 |
| AI-Stance time | 1.526 | 0.132 | 2.778 | 1.805 | 257 |
| AI-SL | 1.080 | 0.363 | 0.002 | 0.585 | 257 |
| TO | **3.987** | 0.340 | 2.563 | 0.052 | 257 |
| HS | **12.692** | **27.272** | **10.257** | **3.931** | 257 |
| CV-TO | **17.831** | **32.390** | **44.736** | **3.902** | 267 |
| CV-HS | **4.144** | **6.180** | 0.009 | 1.050 | 256 |

The Generalized Linear Mixed Model was used to analyze the effects of group, task, group* task.The models were controlled by gender, age, height, weight, education level, score of Mini-Mental State Examination and Hamilton Depression Rating Scale. *P* < 0.05 were considered statistically significant and highlighted in bold. ref, reference; Group*Task: the interaction between group and task; ST, single task; GV, gait velocity; SL, stride length; TO, toe-off angle; HS, heel strike angle; CV, coefficient of variation; AI, asymmetry index; Df, degree of freedom.

Supplemental Table 5. Receiver operating characteristics analysis for gait parameters distinguishing the individuals with early stage Parkinson’s Disease and health controls

|  | AUC | 95%CI | *P* | Sensitivity | Specificity | Optimum threshold |
| --- | --- | --- | --- | --- | --- | --- |
| ST-SL | 0.823 | 0.746-0.895 | <0.001 | 0.680 | 0.854 | 1.083 |
| ST-HS | 0.796 | 0.715-0.877 | <0.001 | 0.711 | 0.805 | 30.025 |
| ST-GV | 0.760 | 0.667-0.851 | <0.001 | 0.670 | 0.780 | 0.960 |
| ST-TO | 0.727 | 0.636-0.818 | <0.001 | 0.701 | 0.707 | 45.125 |
| ST-Swing time | 0.675 | 0.584-0.767 | 0.001 | 0.546 | 0.756 | 0.403 |
| ST-AI-SL | 0.673 | 0.573-0.773 | 0.001 | 0.794 | 0.512 | 16.404 |
| ST-Stance phase | 0.666 | 0.562-0.777 | 0.002 | 0.567 | 0.732 | 64.172 |
| ST-CV-HS | 0.661 | 0.555-0.767 | 0.003 | 0.750 | 0.585 | 22.163 |
| ST-CV-SL | 0.659 | 0.561-0.757 | 0.003 | 0.920 | 0.371 | 23.984 |
| ST-Combination | 0.869 | 0.811-0.927 | <0.001 | 0.708 | 0.927 | 0.773 |
| Adj ST-Combination | 0.855 | 0.791-0.919 | <0.001 | 0.876 | 0.707 | 0.565 |
| DT-SL | 0.836 | 0.769-0.903 | <0.001 | 0.784 | 0.780 | 1.083 |
| DT-HS | 0.830 | 0.757-0.903 | <0.001 | 0.732 | 0.829 | 28.700 |
| DT-GV | 0.795 | 0.709-0.881 | <0.001 | 0.711 | 0.756 | 0.885 |
| DT-TO | 0.756 | 0.671-0.842 | <0.001 | 0.608 | 0.854 | 41.250 |
| DT-Stance phase | 0.706 | 0.611-0.801 | <0.001 | 0.557 | 0.829 | 65.476 |
| DT-CV-SL | 0.683 | 0.587-0.779 | 0.001 | 0.794 | 0.512 | 19.402 |
| DT-AI-SL | 0.677 | 0.575-0.779 | 0.001 | 0.649 | 0.634 | 18.146 |
| DT-CV-TO | 0.667 | 0.567-0.767 | 0.002 | 0.835 | 0.512 | 17.411 |
| DT-CV-HS | 0.654 | 0.552-0.756 | 0.004 | 0.619 | 0.683 | 25.755 |
| DT-AI-Swing time | 0.654 | 0.550-0.758 | 0.004 | 0.639 | 0.683 | 18.184 |
| DT-CV-Swing time | 0.625 | 0.525-0.725 | 0.021 | 0.711 | 0.512 | 20.263 |
| DT-Combination | 0.909 | 0.859-0.959 | <0.001 | 0.897 | 0.829 | 0.599 |
| Adj DT-Combination | 0.869 | 0.806-0.933 | <0.001 | 0.825 | 0.805 | 0.626 |
| ST& DT-Combination | 0.924 | 0.877-0.971 | <0.001 | 0.927 | 0.829 | 0.569 |
| Adj ST& DT-Combination | 0.886 | 0.829-0.943 | <0.001 | 0.876 | 0.780 | 0.611 |

AUC,area under the curve; CI, confifidence interval; ST, single task; DT, dual task; GV, gait velocity; SL, stride length; ST, stride time; TO, toe-off angle; HS, heel strike angle; CV, coefficient of variation; AI, asymmetry index. ST-Combination, receiver operating characteristics (ROC) analysis for the combination of GV, SL, TO, HS, swing time, stance phase,CV-HS, CV-SL, and AI-SL under ST; Adj ST-Combination, ROC analysis for the combination of GV, SL, swing time, stance phase, and HS under ST. DT-Combination, ROC analysis for the combination of GV, SL, TO, HS, stance phase,CV-SL, AI-SL, CV-TO, CV-HS, CV-Swing time and AI-Swing time under DT; Adj DT-Combination, ROC analysis for the combination of GV, SL, stance phase, and HS under DT. ST&DT-Combination, ROC analysis for the combination of all gait parameters with diagnostic values under ST and DT. Adj ST& DT-Combination, ROC analysis for the combination of ST-GV, ST-SL, ST-swing time, ST-stance phase, ST-HS, DT-GV, DT-SL, DT-stance phase, and DT-HS.

Supplemental Table 6. Compare the receiver operating characteristics curves for the diagnosis of early-stage Parkinson’s Disease

| ROC curve1 | ROC curve2 | z | *P* |
| --- | --- | --- | --- |
| ST-GV | DT-GV | -1.494 | 0.135 |
| ST-SL | DT-SL | -0.663 | 0.507 |
| ST-TO | DT-TO | -1.572 | 0.116 |
| ST-HS | DT-HS | -1.896 | 0.058 |
| ST-Stance phase | DT-Stance phase | -1.445 | 0.148 |
| ST-AI-SL | DT-AI-SL | -0.06 | 0.952 |
| ST-CV-SL | DT-CV-SL | -0.386 | 0.699 |
| ST-CV-HS | DT-CV-HS | -0.164 | 0.87 |
| ST-Combination | DT-Combination | -1.839 | 0.066 |
| ST-Combination | ST& DT-Combination | -2.340 | 0.019 |
| Adj ST-Combination | Adj DT-Combination | -0.791 | 0.429 |
| Adj ST-Combination | Adj ST& DT-Combination | -1.563 | 0.118 |
| Adj ST-Combination | ST-Combination | 1.143 | 0.253 |
| Adj DT-Combination | DT-Combination | 1.812 | 0.07 |
| Adj ST&DT-Combination | ST& DT-Combination | -1.813 | 0.07 |

The area under the curve (AUC) was compared using the bootstrap method with 2000 iterations.AUC,area under the curve; CI, confifidence interval; ST, single task; DT, dual task; GV, gait velocity; SL, stride length; ST, stride time; TO, toe-off angle; HS, heel strike angle; CV, coefficient of variation; AI, asymmetry index. ST-Combination, receiver operating characteristics (ROC) analysis for the combination of GV, SL, TO, HS, swing time, stance phase,CV-HS, CV-SL, and AI-SL under ST; Adj ST-Combination, ROC analysis for the combination of GV, SL, swing time, stance phase, and HS under ST. DT-Combination, ROC analysis for the combination of GV, SL, TO, HS, stance phase,CV-SL, AI-SL, CV-TO, CV-HS, CV-Swing time and AI-Swing time under DT; Adj DT-Combination, ROC analysis for the combination of GV, SL, stance phase, and HS under DT. ST&DT-Combination, ROC analysis for the combination of all gait parameters with diagnostic values under ST and DT. Adj ST& DT-Combination, ROC analysis for the combination of ST-GV, ST-SL, ST-swing time, ST-stance phase, ST-HS, DT-GV, DT-SL, DT-stance phase, and DT-HS.
